# The Magnitude Effect on Tactile Spatial Representation: The Spatial–Tactile Association for Response Code (STARC) Effect

**DOI:** 10.3389/fnins.2020.557063

**Published:** 2020-09-29

**Authors:** Alice Bollini, Claudio Campus, Davide Esposito, Monica Gori

**Affiliations:** ^1^Unit for Visually Impaired People, Istituto Italiano di Tecnologia, Genoa, Italy; ^2^DIBRIS, Università di Genova, Genoa, Italy

**Keywords:** spatial reference frame, mental magnitude line, Spatial-Tactile Association for Response Code, spatial S-R compatibility, magnitude S-R compatibility

## Abstract

The human brain uses perceptual information to create a correct representation of the external world. Converging data indicate that the perceptual processing of, space, and quantities frequently is based on a shared mental magnitude system, where low and high quantities are represented in the left and right space, respectively. The present study explores how the magnitude affects spatial representation in the tactile modality. We investigated these processes using stimulus-response (S-R) compatibility tasks (i.e., sensorimotor tasks that present an association/dissociation between the perception of a stimulus and the required action, generally increasing/decreasing accuracy and decreasing/increasing reaction times of the subject). In our study, the participant performed a discrimination task between high- and low-frequency vibrotactile stimuli, regardless of the stimulation’s spatial position. When the response code was incompatible with the mental magnitude line (i.e., left button for high-frequency and right button for low-frequency responses), we found that the participants bypassed the spatial congruence, showing a magnitude S-R compatibility effect. We called this phenomenon the Spatial–Tactile Association of Response Codes (STARC) effect. Moreover, we observed that the internal frame of reference embodies the STARC effect. Indeed, the participants’ performance reversed between uncrossed- and crossed-hands posture, suggesting that spatial reference frames play a role in the process of expressing mental magnitude, at least in terms of the tactile modality.

## Introduction

To interact with the environment, we must plan and execute actions following sensory inputs and their spatial representations. Behavioral performance is better when the stimulus (sensory input) and the response (action) share common spatial features; even these features are task irrelevant. This phenomenon is the stimulus-response (S-R) compatibility effect, which occurs when a stimulus and response action occupy the same spatial position. The Simon effect (Simon and Small, 1969) is a particular case of S-R compatibility wherein participants respond to a non-spatial feature of the left and right stimuli (like color or shape) and their performance is better when the stimulus occupies the same location of the response effectors (hands or button keys)—that is, when the stimulus is spatially congruent with the response. S-R compatibility is not only for explicit spatial locations but also for representational space, where irrelevant spatial information is implicit. This is the case of the spatial–numerical association of response codes or the SNARC effect (Dehaene et al., 1990, 1993). In the SNARC effect, a response to a small number is faster with left effectors while responses to large numbers are faster with right effectors. This effect has led researchers to hypothesize that there exists a mental number line (MNL). This line is internal and spatially organized, as a horizontal linear continuum, with larger numerical magnitudes that are typically located to the right on the line (Dehaene, 1992), at least in Western cultures. Therefore, a key in the left space must respond to a small-number stimulus (or a large number with a key in the right space), it is magnitude congruent to the position of the response. Otherwise, it is magnitude incongruent. After the demonstration of the SNARC effect, many researchers have investigated representational space and mental magnitude (i.e., the mental representation of countable and uncountable quantity) (Gallistel and Gelman, 2000). Specifically, there has been work regarding influence on the horizontal space in S-R compatibility, and how different features become affected. These studies found that not only numbers are represented in a left-to-right fashion, as in the SNARC effect, but also auditory pitch [SMARC effect (Rusconi et al., 2006)], time [STEARC effect (Ishihara et al., 2008)], linguistic markedness, [MARC effect (Nuerk et al., 2004)], size (Ren et al., 2011; Wühr and Seegelke, 2018), and quantity [SQUARC effect (Walsh, 2003; Kirjakovski and Utsuki, 2012] showed a similar representation. For simplicity, we defined all these effects as magnitude S-R compatibility effects (where there is a relationship between magnitude and response mapping). These effects probably reflect the mapping of perceptual stimuli with the response in physical space, built sharing a common mental spatial representation of magnitude. In literature, a common processing mechanism has been proposed where a generalized mental magnitude system would elaborate sensory information about space, time, and numbers (Walsh, 2003; Bueti and Walsh, 2009), which are all necessary for actions. According to the authors, this generalized mental magnitude system is not limited to a single sense modality. Instead, it is based on a general amodal processing mechanism. Indeed, a number of studies have demonstrated magnitude S-R compatibility effects on continue and discrete quantities in both auditory and visual domains (for a recent review, see Macnamara et al., 2018).

While an increasing number of studies investigated this mental magnitude and spatial systems in visual and auditory domains (Wallace, 1971; Roswarski and Proctor, 2000; Wascher et al., 2001; Phillips and Ward, 2002; Roder et al., 2007; Crollen et al., 2017; Ruzzoli and Soto-Faraco, 2017), only a few studies have examined the tactile mental magnitude effect. The few studies that have involved tactile modality are based on a digital representation of the number (Brozzoli et al., 2008; Krause et al., 2013). Such studies have used tactile modality to investigate the relationship between number representation and finger counting using paradigms as numerical distance effect (Krause et al., 2013) or number-based attentional cueing (Brozzoli et al., 2008). These investigate a body-based representation of numbers rather than a possible magnitude effect in tactile modality.

The literature has demonstrated, in several experimental contexts, an association between mental magnitude and space. Then, a reference frame is needed to spatially organize the mental representation of the magnitude information (Gevers and Lammertyn, 2005). However, its characterization is still unclear (Viarouge et al., 2014; Weis et al., 2018). Specifically, the question is whether the spatial-magnitude effect is embodied in an internal, body-centered frame or in an external, object-centered frame. Recently, Viarouge et al. (2014) proposed that magnitude activates an external or internal frame in accordance with experimental setups. This assumption implies that, as the mental magnitude system is amodal, and therefore, the sensory mode itself may trigger the spatial frame activated during the magnitude S-R compatibility task. In fact, it has been demonstrated in the literature that spatial S-R compatibility tasks activate a specific spatial frame depending on the sensory mode of the experimental inputs (Ruzzoli and Soto-Faraco, 2017). Vision and hearing rely more on an external frame, while touch relies more on an internal frame.

In this context, we wish to investigate how mental magnitude affects a spatial S-R compatibility task such as the Simon task in the tactile modality. Specifically, we want to see if magnitude S-R compatibility is strong enough to reverse the effect of spatial S-R compatibility in a case of conflict between mapping code.

We hypothesize that if the mental magnitude affects spatial representation in the tactile modality, then in the scenario that the response code is misaligned, the spatial S-R compatibility effect should reverse. Moreover, we want to study the role of the spatial reference frame in tactile magnitude by crossing the hands over the body midline. We also hypothesize that, if the magnitude S-R compatibility is embodied in an external frame, the hands’ posture should not affect the performance. Otherwise, in the case of an internal frame, the congruency effect should reverse.

## Materials and Methods

### Participants

**FIGURE 1:**
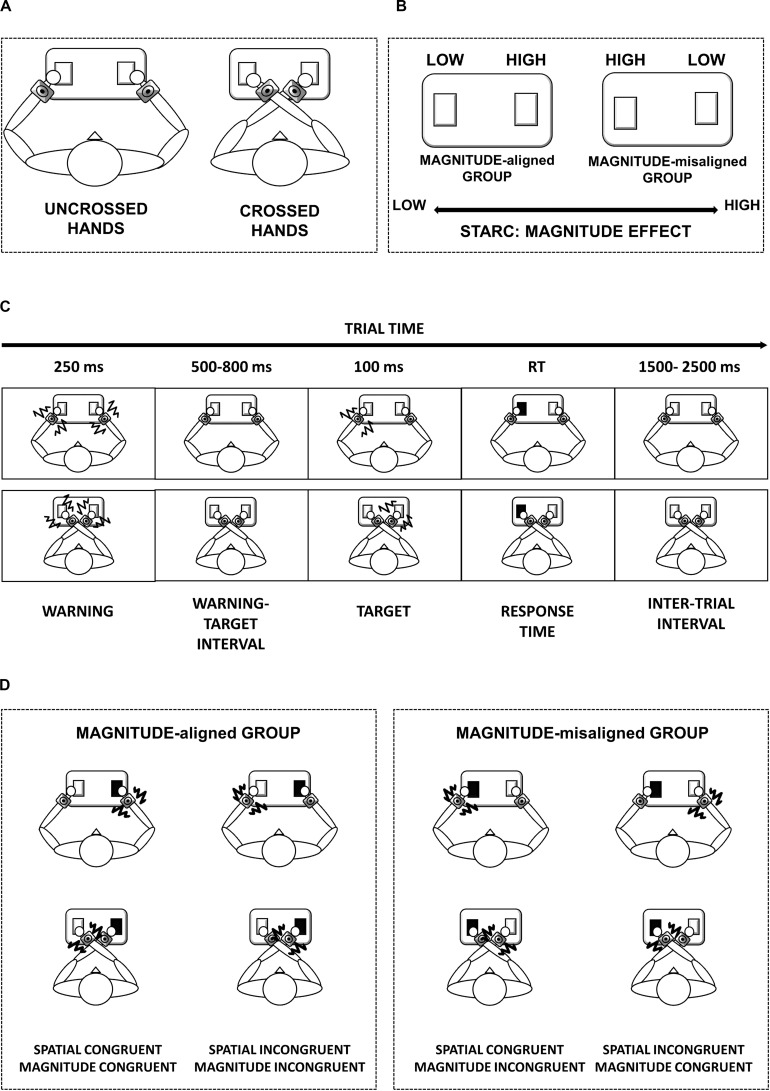
Schematic representation of task setup and procedure. Panel **(A)** represents the two main conditions: uncrossed- and crossed-hands posture. Panel **(B)** represents how we created the two groups: we divided the participants according to response code mapping. Panel **(C)** represents the time window of the trial in uncrossed-hands posture (top) and crossed-hands posture (bottom). Panel **(D)** represents the schema of our experimental conditions in case of high-frequency stimulus in the two groups.

The current study enrolled 32 participants [21 F, aged (M = 26.7 ± SD = 6.5) years, 5 left handed]. They all provided written informed consent in accordance with the Declaration of Helsinki. The ethics committee approved the study of the local health service (Comitato Etico, ASL3 Genovese, Italy). The participants performed a discrimination task between high- and low-frequency vibrotactile stimulations, regardless of the position of the stimuli. To disentangle spatial and magnitude S-R compatibility effects, the study randomly assigned subjects to two groups according to response code mapping (see Figure 1B). We adopted a between-subject design to give each subject a unique set of instructions, thereby preventing subjects from adapting to a certain response code.

The MAGNITUDE-aligned group [11 F, aged (25.7 ± 4.4) years], where the instruction followed the mental magnitude line (i.e., low quantities on the left and high quantities on the right space) and was congruous with the spatial S-R compatibility (like Simon task). While in the MAGNITUDE-misaligned group [10 F, aged (27.6 ± 8.1) years], the instruction was opposite to the mental magnitude. Accordingly, magnitude S-R compatibility was incongruous with the Simon task’s spatial congruency. The MAGNITUDE-aligned group had to press the left key for the low-frequency stimulation and the right key for the high-frequency stimulation. This was *vice versa* for the MAGNITUDE-misaligned group. In this way, for the MAGNITUDE-misaligned group, we create a conflict between the congruency of spatial and magnitude representation (see Figure 1B).

### Apparatus and Stimuli

We ran our experiment using Psychtoolbox-3 (Kleiner et al., 2007) on MatLab^®^ 2018b. Tactile stimuli were vibrotactile stimulations delivered through two modules of MSI Caterpillar (Gori et al., 2014, 2019; Vercillo and Gori, 2015). The stimuli were positioned on the right and left wrists of the participants and fixed using an elastic bandage. The tactile target stimuli were two vibrations lasting 100 ms (stimulus 1 low-frequency stimulation, 60 Hz, 2 V; stimulus 2 high-frequency stimulation, 120 Hz, 3 V), and they can appear either on the left or right wrist. The warning vibration had an intermediate amplitude and frequency between stimulus 1 and 2 and presented simultaneously on both wrists.

We chose the stimulus values based on a pilot study (*n* = 10) to have an accuracy level of around 85%. This was meant to maintain a high level of attention for the entire task duration. We collected the responses using a push-button panel placed in front of participants’ bodies and 14 cm away from the body midline in the left and right hemispaces.

### Procedure

In the study, the participants sat at a table facing straight ahead with their hands on the push-button panel. They were instructed to look at a fixation point that was 95 cm away and central to their position for the whole duration of the task. Each trial began with a warning that lasted for 250 ms, followed by a random delay between 500 and 800 ms. After the delay, the target appeared for 100 ms in the left or right hemispaces. The study asked participants to respond as fast as they possibly could to the frequency of the stimuli (high or low), specifically by pressing the left or right buttons of the push-button panel, regardless of stimulus location. The trials with response times that were over 2.5 s were considered null. The next trial started with a 1.5–2.5-s delay after the response on the preceding trial (see Figure 1C). Each participant performed four blocks of 60 trials, with two for each hands position (order of uncrossing and crossing position were counterbalanced among participants) (see Figure 1A). We calculated the sample size based on samples in previous related stimulus-response studies (Notebaert et al., 2006; Nishimura and Yokosawa, 2009).

### Data Analysis

Spatial S-R congruency was coded based on the relationship between push-button (left/right) and stimulus position. To classify the data, we rated a trial as spatially congruent if the stimulus position matched the push button (left/right) that was necessary for providing the correct response regardless of the arm posture (crossed/uncrossed) (see Figure 1D). For example, in the case of MAGNITUDE-misaligned group instructions (“for higher stimuli, push the left button”), when the hands were crossed, spatial congruent stimulus occurred on the left hand that was on the right space.

For each participant, we excluded all responses that were 2.5 standard deviations above or below the individual mean from the analyses (average, 3.3%; SD, 0.92). We calculated accuracy as the percentage of correct responses and reaction times using the mean of correct answers reaction times.

To avoid a conflict between accuracy and speed scores, as well as to avoid contradictory conclusions, we implemented an integrated speed and accuracy measure. Linear-integrated speed-accuracy score (LISAS) (Vandierendonck, 2017) has been demonstrated to detect effects present in either speed or accuracy, and the correct rate score was efficient in signaling a larger number of strong effects that were unsupported by the speed and accuracy data alone (Vandierendonck, 2017, 2018). LISAS is defined as

(1) $$\text{LISAS}={\text{RT}}_{\text{cond}}+{\text{PE}}_{\text{cond}}\times \frac{\sigma \,{\text{RT}}_{\text{tot}}}{\sigma \,{\text{PE}}_{\text{tot}}}$$

where RT_cond_ is the participant’s mean RT in a condition, PE_cond_ is the participant’s proportion of errors in the same condition, σRT_tot_ is the participant’s overall RT standard deviation in all conditions, and σPE_tot_ is the participant’s overall PE standard deviation, in all conditions. In this way, the errors are weighted with the ratio of the RT and PE standard deviations, so a similar weight of the two components (RT and PE) is achieved (Vandierendonck, 2018). Higher scores on LISAS indicate worse performance (i.e., slower and less accurate) and *vice versa*.

To test the change in spatial representation, we separately fit a linear mixed-effects model to each collected response (LISAS, reaction times, and accuracy scores), using effects coding to describe the factors’ levels (Davis, 2010) and REML as convergence criteria. We analyzed also reaction times and accuracy scores for the sake of completeness and reproducibility with past literature. The fixed effects of the model were the between-subject factor “group” (MAGNITUDE-aligned and MAGNITUDE-misaligned); the within-subject factor “hands posture” (uncross and cross); the within-subject factor “spatial-congruency” (congruent and incongruent); and all the respective interactions. The variability of the within-subject effects was taken into account by modeling them as random intercepts nested within the participant factor. In Wilkinson’s notation (Wilkinson and Rogers, 1973), this model is described by the formula:

RESPONSE∼Group×Hands⁢Posture×C⁢o⁢n⁢g⁢r⁢u⁢e⁢n⁢c⁢y+(1|participant)+(1|HandsPosture:participant)+(1|Congruency:participant)

where RESPONSE can be LISAS, reaction times, or accuracy scores. To evaluate the significance of the model effects, we performed *t* tests on the model estimates using Kenward-Roger’s degrees of freedom approximation (Luke, 2017). The *post hoc* tests were performed on the levels of the highest significant interaction estimate and Bonferroni correction. An additional set of contrasts was planned to directly evaluate the differences between the congruent and incongruent levels of the “spatial-congruency” factor (i.e., the S-R compatibility effect under investigation), or Δ-spatial, per each combination of group and hands posture levels. The analyses were made using R (R Development Core Team, 2011). The model fitting was done using the package *lme4* (Bates et al., 2007), the *t* tests on the fixed effects were done using the package *lmerTest* (Kuznetsova et al., 2017), amd finally, we conducted the *post hoc* comparisons and the planned contrasts using the package *emmeans* (Lenth et al., 2020).

## Results

**FIGURE 2:**
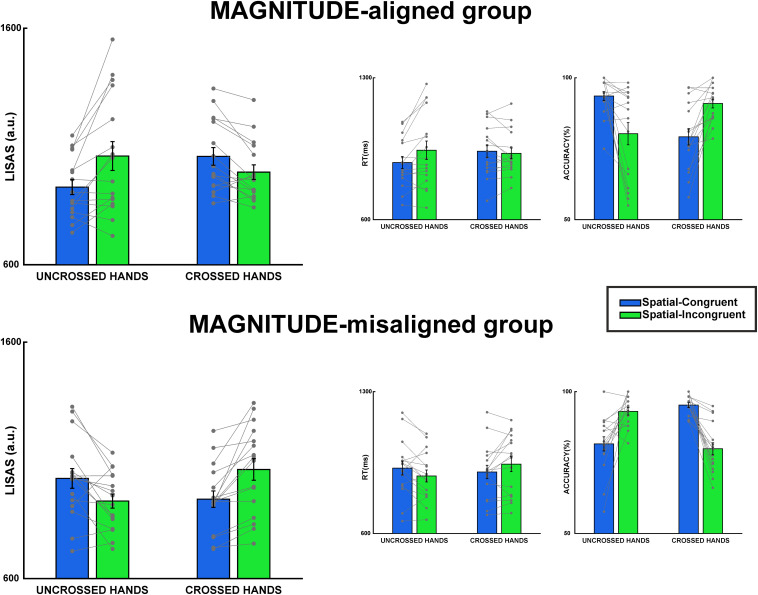
Results of MAGNITUDE-aligned and MAGNITUDE-misaligned groups in Tactile S-R task. The first panel on the left represents LISAS scores for uncrossed and crossed hands. The middle panel represents results on reaction times, and the third panel on the right represents accuracy results. Error bars represent the standard error of the mean (SEM), gray points single-subject performance.

The participants have a mean average performance of 86.28% and a mean reaction time of 928.04 ms (Figure 2, middle and right panels). For all tests, non-significant Levene’s test and Shapiro-Wilk’s test confirmed that assumptions of homogeneity and normality of variance were met.

The fixed-effects estimates of the linear mixed-effect model on the LISAS scores revealed a significant all-factor interaction, *t*(30) = 7.446, *p* < 0.001, Cohen’s *d* = 1.57 (Figure 2 and Table 1). Wealso identified similar interactions for reaction times [*t*(30) = 4.224, *p* < 0.001, Cohen’s *d* = 1.09] and accuracy [*t*(30) = -9.485, *p* < 0.001, Cohen’s *d* = -1.99] (see Table 1, second and third panels).

**TABLE 1:** Outcomes of the linear mixed-effects model for LISAS, reaction times (RT), and accuracy (ACC).

	Estimate	SE	*df*	*t* value	*p*-value
**LISAS**					
*(Intercept)*	998.264	26.774	30	37.284	<0.0001
Group	–11.139	26.774	30	–0.416	0.680
Hands	–13.397	7.038	30	–1.903	0.067
Congruency	–11.871	7.038	30	–1.687	0.102
Group:Hands	2.054	7.038	30	0.292	0.772
Group:Congruency	4.375	7.038	30	0.622	0.539
Hands:Congruency	2.866	7.038	30	0.407	0.687
Group:Hands:Congruency	52.407	7.038	30	7.446	<0.0001
**RT**					
*(Intercept)*	917.657	23.291	30	39.399	<0.0001
Group	–4.576	23.291	30	–0.196	0.846
Hands	–9.970	4.392	30	–2.270	0.031
Congruency	–6.128	4.681	30	–1.309	0.200
Group:Hands	0.189	4.392	30	0.043	0.966
Group:Congruency	6.286	4.681	30	1.343	0.189
Hands:Congruency	0.767	4.392	30	0.175	0.862
Group:Hands:Congruency	18.550	4.392	30	4.224	<0.0001
**ACC**					
*(Intercept)*	86.714	1.171	30	74.080	<0.0001
Group	0.723	1.171	30	0.618	0.541
Hands	0.407	0.683	30	0.596	0.556
Congruency	0.683	0.683	30	0.999	0.326
Group:Hands	–0.525	0.683	30	–0.768	0.448
Group:Congruency	0.301	0.683	30	0.441	0.662
Hands:Congruency	–0.227	0.683	30	–0.332	0.742
Group:Hands:Congruency	–6.480	0.683	30	–9.485	<0.0001

**FIGURE 3:**
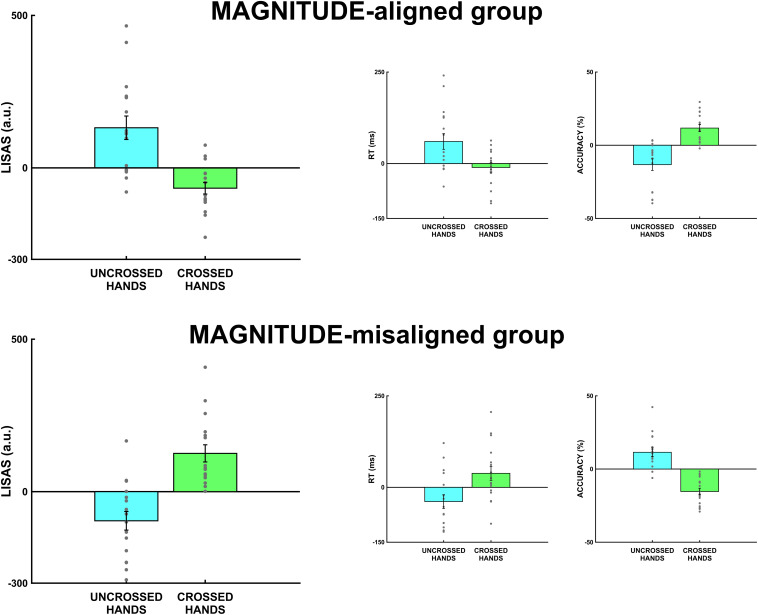
Spatial-Δ S-R (spatial-incongruent MINUS spatial-congruent) for MAGNITUDE-aligned and MAGNITUDE-misaligned groups. The first panel on the left represents LISAS scores for uncrossed and crossed hands. The middle panel represents results on reaction times, while the third panel on the right represents accuracy results. Error bars represent the standard error of the mean (SEM), gray points single-subject performance.

For brevity, we will only discuss *post hoc* tests on LISAS scores. The *post hoc* tests on reaction times and accuracy showed similar results, as shown in Table 2. The *post hoc* tests on LISAS scores revealed that the S-R compatibility effect (incongruent ≠congruent) was present in both groups: in the MAGNITUDE-aligned group in the uncrossed-hands posture, *t*(60) = 4.67, *p*_bonf_ < 0.001, Cohen’s *d* = 1.65, but not in the crossed-hands posture, *t*(60) = -2.36, *p*_bonf_ = 0.08, Cohen’s *d* = -0.84. On the other hand, the MAGNITUDE-misaligned group was present in both hands posture, but with opposite sign (i.e., uncrossed-hands posture, *t*(60) = -3.39, *p*_bonf_ < 0.005, Cohen’s *d* = -1.2, crossed-hands posture, *t*(60) = 4.46, *p*_bonf_ < 0.001, Cohen’s *d* = 1.58). The planned contrasts on Δ-spatial revealed that the two groups differed both in uncrossed (*t*(60) = -5.47, *p*_bonf_ < 0.001, Cohen’s *d* = -2.74) and crossed [*t*(60) = 5.06, *p*_bonf_ < 0.001, Cohen’s *d* = 2.53] hands posture. Furthermore, observed that in the MAGNITUDE-aligned group, the Δ-spatial was different between uncrossed and crossed conditions, *t*(30) = 4.98, *p*_bonf_ < 0.001, Cohen’s *d* = 2.49. In the MAGNITUDE-misaligned group, the difference between uncrossed and crossed Δ-spatial was opposite *t*(30) = -5.55, *p*_bonf_ < 0.001, Cohen’s *d* = -2.78. As illustrated in Figure 3, there is a clear opposite spatial S-R congruency effect (i.e., magnitude S-R congruency effect) for the MAGNITUDE-misaligned group in the uncrossed-hands condition. Interestingly, the S-R congruency effect is observably inverted for both groups in the crossed-hands posture condition.

**TABLE 2:** *Post hoc* tests for LISAS, reaction times (RT), and accuracy (ACC).

Contrast	Group	Hands posture	Estimate	SE	*df*	*t* ratio	*p*_bonf_
**LISAS**							
Incongruent – Congruent	MAGNITUDE-misaligned	Uncrossed	−95.552	28.153	60	−3.394	0.0049
Incongruent – Congruent	MAGNITUDE-aligned	Uncrossed	131.574	28.153	60	4.673	0.0001
Incongruent – Congruent	MAGNITUDE-misaligned	Crossed	125.538	28.153	60	4.459	0.0001
Incongruent – Congruent	MAGNITUDE-aligned	Crossed	−66.590	28.153	60	−2.365	0.0851
**RT**							
Incongruent – Congruent	MAGNITUDE-misaligned	Uncrossed	−38.948	18.154	60	−2.145	0.1440
Incongruent – Congruent	MAGNITUDE-aligned	Uncrossed	60.393	18.154	60	3.327	0.0060
Incongruent – Congruent	MAGNITUDE-misaligned	Crossed	38.319	18.154	60	2.111	0.1559
Incongruent – Congruent	MAGNITUDE-aligned	Crossed	−10.737	18.154	60	−0.591	1
**ACC**							
Incongruent – Congruent	MAGNITUDE-misaligned	Uncrossed	11.447	2.733	60	4.188	<0.0001
Incongruent – Congruent	MAGNITUDE-aligned	Uncrossed	−13.270	2.733	60	−4.855	<0.0001
Incongruent – Congruent	MAGNITUDE-misaligned	Crossed	−15.383	2.733	60	−5.629	<0.0001
Incongruent – Congruent	MAGNITUDE-aligned	Crossed	11.744	2.733	60	4.297	<0.0001

## Discussion

This study firstly aimed to investigate the effect of the mental magnitude line on a tactile S-R compatibility task. We hypothesize that if the mental magnitude affects spatial representation in the tactile modality, then, when the response code is misaligned, the spatial S-R compatibility effect should reverse, affected by the internal frame. As predicted, we demonstrated in tactile modality, for the first, the presence of a magnitude-congruency effect on horizontal response position and that the mental magnitude effects itself can overcome spatial S-R compatibility. In particular, our first result was a reverse spatial S-R compatibility in the group with a misaligned magnitude response mapping, where participants showed better performance in the spatial incongruent than congruent trials. The reason why this happened is that, even if the stimulus had the same spatial position of the response, the participant followed their mental magnitude representation. This led to a magnitude-congruency effect with faster and more accurate performance for low-frequency stimuli responses on the right space and high-frequency stimuli responses in the left space, such as spatial incongruent trial rather than the opposite. These results suggest that, in tactile modality, mental magnitude representation activated the magnitude-congruency effect more than the spatial-congruency—that is, the Spatial–Tactile Association of Response Codes (STARC) effect.

While studies have increasingly investigated numerosity processing and finger-number association, few have examined the association of the tactile modality with space (i.e., low/high frequencies associated with left/right spaces, respectively)—that is, until now. A fact that has emerged from studies regarding numerosity and subitizing (Riggs et al., 2006; Brozzoli et al., 2008; Plaisier et al., 2009; Plaisier and Smeets, 2011; Krause et al., 2013) is that touch and vision share the same numerosity representation. Here, to investigate the effect of mental magnitude representation on the spatial S-R compatibility effect, we insert conflicts into two S-R congruency tasks.

We found a reverse spatial S-R compatibility (also known as reverse Simon effect) resulting from the STARC effect’s interference. Additionally, this result agrees with previous works. The reverse spatial S-R compatibility has been demonstrated in many situations, such as in visual Simon tasks, with an incompatible color-mapping instruction (press red response key on green stimulus and *vice versa*) (Hedge and Marsh, 1975; Lu and Proctor, 1995) or after a long practice with incompatible mapping instruction in visual tasks (i.e., practice and Simon tasks with opposite response code) (Proctor and Lu, 1999; Proctor and Marble, 2000; Tagliabue et al., 2000). A reverse spatial S-R compatibility effect has also been found during SNARC effect manipulation (Notebaert et al., 2006). Notebaert et al. (2006) identified this effect in alternating trials of spatial-congruency with number-congruency task with aligned and non-aligned instructions. We can observe the same effect in our experiment, in which the incompatible magnitude mappings change the associations between spatial representation and spatial response code in favor of the STARC effect (Figure 2, bottom panel). Our results reveal the existence of a map that translates the mental magnitude, a non-spatial domain, to a mental spatial continuum (from left-to-right) that interacts directly with the spatial representation, at least for the tactile modality. However, we must underline that our reaction times were long (because of task difficulty, i.e., accuracy level 85%) and this fact probably increased the opportunity for the participant to follow the magnitude-congruency effect rather than the spatial-congruency effect. Indeed, some researchers have demonstrated that, in the spatial S-R compatibility tasks, as RTs increase, the spatial-congruency effect decreases while the magnitude-congruency effect increases (Mapelli et al., 2003; Gevers et al., 2005). This is the case in experiments where Simon and SNARC effects interact (i.e., parity judgment, by pressing a left or right key, where the numbers were presented to either the left or right side of fixation). Nevertheless, Andres et al. (2008) have proposed that the mental magnitude information becomes spatially relevant just when it has been mapped somehow in the bodily experience. In our manipulation, this fills an important role because we used tactile stimuli that are relevant for body experience, as the tactile modality is embodied in body-related coordinates (internal reference). In our manipulation, we found an interaction between magnitude and spatial congruency; the existence of this interaction implies that the two different sources of spatial information are somehow combined before resulting in a univocal spatial representation.

As previous literature extensively shows (Vandierendonck, 2017, 2018; Liefooghe and De Houwer, 2018), LISAS is more sensitive and efficient because it integrates two complementary aspects (RT and subjects’ accuracy) into one single score. Our second goal was to investigate the frame of reference in tactile mental magnitude mechanisms. To disentangle external and internal frames, we created a conflict between the frames by crossing one’s hands over the body midline. This manipulation misaligns the effectors with their position in space and the stimuli position. We found a significant interaction between group, congruency, and hands posture. This interaction derived from the opposite direction of the congruency effect when the participants crossed their hands, independent from group belonging, as demonstrated from the opposite sign in our planned contrasts. In other words, the MAGNITUDE-aligned group presented with a reverse Simon effect with crossed hands, and the MAGNITUDE-misaligned group had a normal spatial congruency effect with crossed hands. This result can be interpreted as the dominance of the internal frame where the spatial S-R congruency is between the position of the stimulus and the anatomical location of the responding effector (the hand) because the participants, in tactile tasks, follow body-centered coordinates. Many studies have used crossed hands to test the spatial reference frame during a spatial S-R congruency task, such as Simon task. These studies have tested spatial coordinates in various sensory modalities, such as vision (Wallace, 1971; Roswarski and Proctor, 2000; Wascher et al., 2001; Phillips and Ward, 2002; Ruzzoli and Soto-Faraco, 2017), hearing (Roswarski and Proctor, 2000; Roder et al., 2007; Crollen et al., 2017), and touch (Medina et al., 2014; Ruzzoli and Soto-Faraco, 2017). This demonstrates that visual and audio modalities are based more heavily in external coordinates, while tactile ones rely on internal coordinates. Instead, regarding the association of mental magnitude and spatial coordinates, a question remains as to which reference frames are involved. The major part of the literature has focused on the SNARC effect to date (for a review, see Viarouge et al., 2014).

Here, we have demonstrated that an internal frame of reference triggered the STARC effect. These results are in line with our recent observations (Viarouge et al., 2014; Mourad and Leth-Steensen, 2017), in which we modulated the instructions to rely either on the external frame (with object-based instructions) or on the internal frame (hand-based instructions). These studies have generally concluded the existence of a hierarchical spatial frame that would trigger internal or external spatial frames of reference based on a single experimental context. Indeed, we could have found an internal influence on magnitude because we used tactile stimuli typically involving active body-centered coordinates. It would be interesting to investigate how crossing the hands influences the magnitude effect in auditory or vision modality when the subjects perform a spatial S-R compatibility task with instructions that create a conflict between magnitude and spatial representations.

In this work, we used a SNARC-like task to investigate the spatial encoding of a tactile stimulus’ magnitude, as well as its interaction with the egocentric internal reference frame. This idea relied on the postulate that all numerosity inputs coming from different modalities are themselves embodied in an amodal general magnitude system (Walsh, 2003). Nonetheless, as posited by Casasanto and Pitt (2019), SNARC-like tasks cannot be used as a test for the general magnitude system: when we are making a magnitude judgment, it is impossible that also the ordinal aspect of the stimuli are not also represented. It is true that the main characteristic of magnitude is being structured as a quantitative, absolute measure; however, it can also be processed as a relative, “more than” vs. “less than” scale, given a reference point. Therefore, it is possible that a general polarity system exists (Proctor and Cho, 2006), and regulates the activation of magnitude or spatial processing during stimulus-response tasks based on an arbitrary pole. Our study does not support any of these mechanistic theories, rather, it provides further evidence for the existence of such class of effects.

In conclusion, we demonstrated that the magnitude-congruency effect is present in the tactile modality, and its impact therein is stronger than the spatial-congruency effect. As a result, in the case of conflict between two S-R compatibility effects, the magnitude-congruency effect prevails. Moreover, we have demonstrated that this effect relies on internal coordinates, considering that the tactile modality is embodied in the internal frame.

## Data Availability Statement

The raw data supporting the conclusions of this article will be made available by the authors, without undue reservation.

## Ethics Statement

The studies involving human participants were reviewed and approved by Comitato Etico, ASL3 Genovese, Italy. The patients/participants provided their written informed consent to participate in this study.

## Author Contributions

AB, MG, CC, and DE conceived the study and designed the experiments. AB and DE carried out experiments and analyzed the data. MG, CC, and AB wrote the manuscript. AB prepared figures. All authors reviewed the manuscript.

## Conflict of Interest

The authors declare that the research was conducted in the absence of any commercial or financial relationships that could be construed as a potential conflict of interest.

## References

[B1] Andres M. Olivier E. Badets A. (2008). Actions, words, and numbers: a motor contribution to semantic processing? *Curr. Dir. Psychol. Sci.* 17 313–317. 10.1111/j.1467-8721.2008.00597.x

[B2] Bates D. Sarkar D. Bates M. D. Matrix L. (2007). The lme4 package. *R Pack. Vers.* 2:74.

[B3] Brozzoli C. Ishihara M. Gobel S. M. Salemme R. Rossetti Y. Farne A. (2008). Touch perception reveals the dominance of spatial over digital representation of numbers. *Proc. Natl. Acad. Sci. U.S.A.* 105 5644–5648. 10.1073/pnas.0708414105 18385382 PMC2291099

[B4] Bueti D. Walsh V. (2009). The parietal cortex and the representation of time, space, number and other magnitudes. *Philos. Trans. R Soc. B Biol. Sci.* 364 1831–1840. 10.1098/rstb.2009.0028 19487186 PMC2685826

[B5] Casasanto D. Pitt B. (2019). The faulty magnitude detector: why SNARC-like tasks cannot support a generalized magnitude system. *Cogn. Sci.* 43:12794. 10.1111/cogs.12794 31621122

[B6] Crollen V. Albouy G. Lepore F. Collignon O. (2017). How visual experience impacts the internal and external spatial mapping of sensorimotor functions. *Sci. Rep.* 7 1–9. 28432316 10.1038/s41598-017-01158-9 PMC5430802

[B7] Davis M. J. (2010). Contrast coding in multiple regression analysis: strengths, weaknesses, and utility of popular coding structures. *J. Data Sci.* 8 61–73.

[B8] Dehaene S. (1992). Varieties of numerical abilities. *Cognition* 44 1–42. 10.1016/0010-0277(92)90049-n 1511583

[B9] Dehaene S. Bossini S. Giraux P. (1993). The mental representation of parity and number magnitude. *J. Exp. Psychol. Gen.* 122 371–396. 10.1037/0096-3445.122.3.371

[B10] Dehaene S. Dupoux E. Mehler J. (1990). Is numerical comparison digital? Analogical and symbolic effects in two-digit number comparison. *J. Exp. Psychol. Hum. Percept. Perform.* 16 626–641. 10.1037/0096-1523.16.3.626 2144576

[B11] Gallistel C. R. Gelman R. (2000). Non-verbal numerical cognition: from reals to integers. *Trends Cogn. Sci.* 4 59–65. 10.1016/s1364-6613(99)01424-2 10652523

[B12] Gevers W. Caessens B. Fias W. (2005). Towards a common processing architecture underlying Simon and SNARC effects. *Eur. J. Cogn. Psychol.* 17 659–673. 10.1080/09541440540000112

[B13] Gevers W. Lammertyn J. (2005). *The Hunt for SNARC.* Available online at: https://biblio.ugent.be/publication/328538/file/6791361 (accessed August 7, 2019) 10.1080/09541440540000112

[B14] Gori M. Bollini A. Maviglia A. Amadeo M. B. Tonelli A. Crepaldi M. (2019). “MSI Caterpillar: An effective multisensory system to evaluate spatial body representation,” in *Proceedings of the 2019 IEEE International Symposium on Medical Measurements and Applications (MeMeA)*, Piscataway, NJ.

[B15] Gori M. Vercillo T. Sandini G. Burr D. (2014). Tactile feedback improves auditory spatial localization. *Front. Psychol.* 5:1121. 10.3389/fpsyg.2014.01121 25368587 PMC4202795

[B16] Hedge A. Marsh N. W. A. (1975). The effect of irrelevant spatial correspondences on two-choice response-time. *Acta Psychol.* 39 427–439. 10.1016/0001-6918(75)90041-4 1199779

[B17] Ishihara M. Keller P. E. Rossetti Y. Prinz W. (2008). Horizontal spatial representations of time: evidence for the STEARC effect. *Cortex* 44 454–461. 10.1016/j.cortex.2007.08.010 18387578

[B18] Kirjakovski A. Utsuki N. (2012). From SNARC to SQUARC: universal mental quantity line? *Int. J. Psychol. Stud.* 4 217–227.

[B19] Kleiner M. Brainard D. Pelli D. G. Ingling A. Murray R. Broussard C. (2007). What’s new in Psychtoolbox-3. *Perception* 36 1–16.

[B20] Krause F. Bekkering H. Lindemann O. (2013). A feeling for numbers: shared metric for symbolic and tactile numerosities. *Front. Psychol.* 4:7. 10.3389/fpsyg.2013.00007 23355831 PMC3554835

[B21] Kuznetsova A. Brockhoff P. B. Christensen R. H. B. (2017). lmerTest Package: tests in linear mixed effects models. *J. Stat. Softw.* 82 1–26.

[B22] Lenth R. Singmann H. Love J. Buerkner P. Herve M. (2020). *Package ‘emmeans.’ R Packag Version 146.*

[B23] Liefooghe B. De Houwer J. (2018). Automatic effects of instructions do not require the intention to execute these instructions. *J. Cogn. Psychol.* 30 108–121. 10.1080/20445911.2017.1365871

[B24] Lu C. H. Proctor R. W. (1995). The influence of irrelevant location information on performance: a review of the Simon and spatial Stroop effects. *Psychon. Bull. Rev.* 2 174–207. 10.3758/bf03210959 24203654

[B25] Luke S. G. (2017). Evaluating significance in linear mixed-effects models in R. *Behav. Res. Methods* 49 1494–1502. 10.3758/s13428-016-0809-y 27620283

[B26] Macnamara A. Keage H. A. D. Loetscher T. (2018). Mapping of non-numerical domains on space: a systematic review and meta-analysis. *Exp. Brain Res.* 236 335–346. 10.1007/s00221-017-5154-6 29279982

[B27] Mapelli D. Rusconi E. Umiltà C. (2003). The SNARC effect: an instance of the Simon effect? *Cognition* 88 B1–B10. 12804817 10.1016/s0010-0277(03)00042-8

[B28] Medina J. McCloskey M. Coslett H. B. Rapp B. (2014). Somatotopic {Representation} of {Location}: {Evidence} {From} the {Simon} {Effect}. *J. Exp. Psychol. Hum. Percept. Perform.* 40 2131–2142. 10.1037/a0037975 25243674 PMC4684707

[B29] Mourad A. Leth-Steensen C. (2017). Spatial reference frames and SNARC. *J. Cogn. Psychol.* 29 113–128. 10.1080/20445911.2016.1249483

[B30] Nishimura A. Yokosawa K. (2009). Effects of laterality and pitch height of an auditory accessory stimulus on horizontal response selection: the Simon effect and the SMARC effect. *Psychon. Bull. Rev.* 16 666–670. 10.3758/pbr.16.4.666 19648450

[B31] Notebaert W. Gevers W. Verguts T. Fias W. (2006). Shared spatial representations for numbers and space: the reversal of the SNARC and the Simon effects. *J. Exp. Psychol. Hum. Percept. Perform.* 32 1197–1207. 10.1037/0096-1523.32.5.1197 17002531

[B32] Nuerk H. C. Iversen W. Willmes K. (2004). Notational modulation of the SNARC and the MARC (linguistic markedness of response codes) effect. *Q. J. Exp. Psychol. Sect. A Hum. Exp. Psychol.* 57 835–863. 10.1080/02724980343000512 15204120

[B33] Phillips J. C. Ward R. (2002). S-R correspondence effects of irrelevant visual affordance: time course and specificity of response activation. *Vis. Cogn.* 9 540–558. 10.1080/13506280143000575

[B34] Plaisier M. A. Bergmann Tiest W. M. Kappers A. M. L. (2009). One, two, three, many - subitizing in active touch. *Acta Psychol.* 131 163–170. 10.1016/j.actpsy.2009.04.003 19460685

[B35] Plaisier M. A. Smeets J. B. J. (2011). Haptic subitizing across the fingers. *Attent. Percept. Psychophys.* 73:1579. 10.3758/s13414-011-0124-8 21479724 PMC3118010

[B36] Proctor R. W. Cho Y. S. (2006). Polarity correspondence: a general principle for performance of speeded binary classification tasks. *Psychol. Bull.* 416–442. 10.1037/0033-2909.132.3.416 16719568

[B37] Proctor R. W. Lu C. H. (1999). Processing irrelevant location information: practice and transfer effects in choice-reaction tasks. *Mem. Cogn.* 27 63–77. 10.3758/bf03201214 10087857

[B38] Proctor R. W. Marble G. (2000). Mixing incompatibly mapped location-relevant trials with location-irrelevant trials?: effects of stimulus mode on the reverse Simon effect. *Psychol. Res.* 64 11–24. 10.1007/s004260000041 11109864

[B39] R Development Core Team (2011). *R: A Language and Environment for Statistical Computing.* Vienna: R Development Core Team.

[B40] Ren P. Nicholls M. E. R. Ma Y.-Y. Chen L. (2011). Size matters: non-numerical magnitude affects the spatial coding of response. *PLoS One* 6:e23553. 10.1371/journal.pone.0023553 21853151 PMC3154948

[B41] Riggs K. J. Ferrand L. Lancelin D. Fryziel L. Dumur G. Simpson A. (2006). Subitizing in tactile perception. *Psychol. Sci.* 17 271–272. 10.1111/j.1467-9280.2006.01696.x 16623680

[B42] Roder B. Kusmierek A. Spence C. Schicke T. (2007). Developmental vision determines the reference frame for the multisensory control of action. *Proc. Natl. Acad. Sci. U.SA.* 104 4753–4758. 10.1073/pnas.0607158104 17360596 PMC1838672

[B43] Roswarski T. E. Proctor R. W. (2000). Auditory stimulus-response compatibility: is there a contribution of stimulus-hand correspondence? *Psychol. Res. Psychol. Forsch.* 63 148–158. 10.1007/pl00008173 10946588

[B44] Rusconi E. Kwan B. Giordano B. L. Butterworth B. Umilta C. (2006). Spatial representation of pitch height?: the SMARC effect spatial representation of pitch height?: the SMARC effect. *Cognition* 99 113–129. 10.1016/j.cognition.2005.01.004 15925355

[B45] Ruzzoli M. Soto-Faraco S. (2017). Modality-switching in the simon task: the clash of reference frames. *J. Exp. Psychol. Gen.* 146 1478–1497. 10.1037/xge0000342 28639796

[B46] Simon J. R. Small A. M. (1969). Processing auditory information: interference from an irrelevant cue. *J. Appl. Psychol.* 53 433–435. 10.1037/h0028034 5366316

[B47] Tagliabue M. Zorzi M. Umiltà C. Bassignani F. (2000). The role of long-term-memory and short-term-memory links in the Simon effect. *J. Exp. Psychol. Hum. Percept. Perform.* 26 648–670. 10.1037/0096-1523.26.2.648 10811168

[B48] Vandierendonck A. (2017). A comparison of methods to combine speed and accuracy measures of performance: a rejoinder on the binning procedure. *Behav. Res. Methods* 49 653–673. 10.3758/s13428-016-0721-5 26944576

[B49] Vandierendonck A. (2018). Further tests of the utility of integrated speed-accuracy measures in Task Switching. *J. Cogn.* 1:8. 10.5334/joc.6 PMC6646946 31517182

[B50] Vercillo T. Gori M. (2015). Attention to sound improves auditory reliability in audio-tactile spatial optimal integration. *Front. Integr. Neurosci.* 9:34. 10.3389/fnint.2015.00034 25999825 PMC4423351

[B51] Viarouge A. Hubbard E. M. Dehaene S. (2014). The organization of spatial reference frames involved in the SNARC effect. *Q. J. Exp. Psychol.* 67 1484–1499. 10.1080/17470218.2014.897358 24571534

[B52] Wallace R. J. (1971). S-R compatibility and the idea of a response code. *J. Exp. Psychol.* 88 354–360. 10.1037/h0030892 5090926

[B53] Walsh V. (2003). A theory of magnitude: common cortical metrics of time, space and quantity. *Trends Cogn. Sci.* 7 483–488. 10.1016/j.tics.2003.09.002 14585444

[B54] Wascher E. Schatz U. Kuder T. Verleger R. (2001). Validity and boundary conditions of automatic response activation in the Simon task. *J. Exp. Psychol. Hum. Percept. Perform.* 27 731–751. 10.1037/0096-1523.27.3.731 11424658

[B55] Weis T. Nuerk H. C. Lachmann T. (2018). Attention allows the SNARC effect to operate on multiple number lines. *Sci. Rep.* 8:13778. 10.1038/s41598-018-32174-y PMC6137054 30214027

[B56] Wilkinson G. N. Rogers C. E. (1973). Symbolic description of factorial models for analysis of variance. *J. Appl. Stat.* 22 392–399. 10.2307/2346786

[B57] Wühr P. Seegelke C. (2018). Compatibility between physical stimulus size and left-right responses: small is left and large is right. *J. Cogn.* 1:17. 10.5334/joc.19 31517191 PMC6634365

